# Exogenous vasopressin dose-dependently modulates gastric microcirculatory oxygenation in dogs via V1A receptor

**DOI:** 10.1186/s13054-019-2643-y

**Published:** 2019-11-12

**Authors:** Richard Truse, Steven Grewe, Anna Herminghaus, Jan Schulz, Andreas P. M. Weber, Tabea Mettler-Altmann, Inge Bauer, Olaf Picker, Christian Vollmer

**Affiliations:** 1 Department of Anesthesiology, Duesseldorf University Hospital, Moorenstr 5, 40225 Duesseldorf, Germany; 2 Institute of Plant Biochemistry, Cluster of Excellence on Plant Sciences (CEPLAS), Heinrich-Heine-University Duesseldorf, Universitätsstr. 1, 40225 Duesseldorf, Germany

**Keywords:** Gastric microcirculation, Vasopressin, V1A receptor

## Abstract

**Background:**

Hypercapnia improves gastric microcirculatory oxygenation (μHbO_2_) and increases vasopressin plasma levels, whereas V1A receptor blockade abolishes the increase of μHbO_2_. The aim of this study was to evaluate the effect of exogenous vasopressin (AVP) in increasing doses on microcirculatory perfusion and oxygenation and systemic hemodynamic variables. Furthermore, we evaluated the role of the vasopressin V1A receptor in mediating the effects.

**Methods:**

In repetitive experiments, six anesthetized dogs received a selective vasopressin V1A receptor inhibitor ([Pmp^1^, Tyr (Me)^2^]-Arg^8^-Vasopressin) or sodium chloride (control groups). Thereafter, a continuous infusion of AVP was started with dose escalation every 30 min (0.001 ng/kg/min–1 ng/kg/min). Microcirculatory variables of the oral and gastric mucosa were measured with reflectance spectrometry, laser Doppler flowmetry, and incident dark field imaging. Transpulmonary thermodilution was used to measure systemic hemodynamic variables. AVP plasma concentrations were measured during baseline conditions and 30 min after each dose escalation.

**Results:**

During control conditions, gastric μHbO_2_ did not change during the course of experiments. Infusion of 0.001 ng/kg/min and 0.01 ng/kg/min AVP increased gastric μHbO_2_ to 87 ± 4% and 87 ± 6%, respectively, compared to baseline values (80 ± 7%), whereas application of 1 ng/kg/min AVP strongly reduced gastric μHbO_2_ (59 ± 16%). V1A receptor blockade prior to AVP treatment abolished these effects on μHbO_2_. AVP dose-dependently enhanced systemic vascular resistance (SVR) and decreased cardiac output (CO). After prior V1A receptor blockade, SVR was reduced and CO increased (0.1 ng/kg/min + 1 ng/kg/min AVP).

**Conclusions:**

Exogenous AVP dose-dependently modulates gastric μHbO_2_, with an increased μHbO_2_ with ultra-low dose AVP. The effects of AVP on μHbO_2_ are abolished by V1A receptor inhibition. These effects are independent of a modulation of systemic hemodynamic variables.

## Background

A relative vasopressin deficiency contributes to the development of vasodilatory shock, e.g., during sepsis [1]. Thus, the Surviving Sepsis Campaign (SSC) with a weak recommendation suggests to add vasopressin to norepinephrine with the intent of raising MAP (mean arterial pressure) or to decrease norepinephrine dosage [2]. Three subtypes of vasopressin receptors, V1A, V1B, and V2 receptors, have been identified. The V1A receptors are found on various cells including vascular smooth muscle cells, the V2 receptor is mainly found in the kidneys [3], and the V1B receptors are predominantly located in the adenohypophysis [4]. Circulating vasopressin, by acting via V1A receptors, is an important backup system for blood pressure control [5]. However, 2 multicenter, randomized clinical trials including 1187 patients with septic shock failed to show a significant difference in mortality rates between patients treated with norepinephrine and patients treated with norepinephrine and vasopressin [6, 7]. In addition, vasopressin may induce exuberant vasoconstriction in the gastrointestinal circulation [8]. The splanchnic circulation is known to be impaired early during compromised circulatory conditions, as the blood flow is redistributed in favor of more vital organs. The gastrointestinal mucosa functions as an effective barrier against bacteria and toxins in the intestinal lumen. A failure of the mucosal barrier function, e.g., because of an impairment in splanchnic microcirculation, is thought to play a pivotal role in the development of sepsis and multiorgan dysfunction [9]. So far, attempts to specifically resuscitate the gut have shown to be unsuccessful [10]. In previous studies, we could show that hypercapnia increased gastric μHbO_2_ in anesthetized dogs [11] and preserved intestinal μHbO_2_ in septic rats [12]. Both effects were abolished during V1A receptor blockade indicating an involvement of the vasopressin system. Hypercapnia has been shown to cause a slight increase in vasopressin plasma concentration [13, 14], whereas administration of 0.03 U/min vasopressin results in a profound rise in vasopressin plasma levels [6]. This suggests that the increase of μHbO_2_ is mediated via V1A receptors due to increase of endogenous vasopressin plasma levels under hypercapnia. It is unclear whether exogenous vasopressin induces the same effect without side effects of hypercapnia. To our knowledge, no dose-response studies exist in a large animal model with the focus on a vasopressin dosage considerably below the clinically used dosage of 0.02–0.04 U/min.

Therefore, the aim of this study was to evaluate the effects of continuously infused vasopressin with a significantly lower dose than used in vasodilatory shock and with the dosage recommended by the SSC in septic shock (0.02–0.04 U/min) on gastric and oral mucosal perfusion and oxygenation in healthy, anesthetized dogs. Selective receptor blockade is used to elucidate the significance of the V1A receptor concerning local mucosal microcirculation.

## Methods

### Animals

The data were derived from repetitive experiments on six dogs (female foxhounds, weighing 28–36 kg). The anatomic conditions in dogs enable the non-traumatic placement of the measuring probe to the gastric mucosa without laparotomy. Additionally, larger blood sample volumes can be collected without hemodynamic side effects. Repetitive experiments are possible which reduce the number of experiments and hence the number of laboratory animals. There is no need to sacrifice the animals at the end of the experiment. Prior to the experiments, access to food was withheld for 12 h with water ad libitum to ensure complete gastric depletion and to avoid changes in mucosal perfusion and oxygenation due to digestive activity and to allow the undisturbed coupling of the measuring probe to the mucosa. Each dog underwent each experimental protocol in a randomized order and served as its own control. The experiments were performed at least 3 weeks apart to prevent carryover effects. The experiments were performed under general anesthesia (induction of anesthesia with 4 mg·kg^−1^ propofol, maintenance with sevoflurane, end-tidal concentration of 3.0% (1.5 minimum alveolar concentration (MAC) for dogs)). The dogs were mechanically ventilated (FiO_2_ = 0.3, tidal volume = 12.5 ml·kg^−1^, a normal tidal volume for dogs [15]) after endotracheal intubation with the respiratory frequency adjusted to achieve normocapnia (end-expiratory carbon dioxide (etCO_2_) = 35 mmHg), verified by continuous capnography (Capnomac Ultima, Datex Instrumentarium, Helsinki, Finland). During baseline conditions, the dogs were placed on their right side and covered with isolating blankets to maintain the body temperature at 37.5 °C (continuous arterial measurement). Throughout the experiments, no additional fluid replacement was carried out to avoid volume effects that could influence tissue perfusion and oxygenation. However, after withdrawal of each blood sample, normal saline was infused at three times the sampling volume to maintain blood volume.

### Measurements

#### Systemic hemodynamic and oxygenation variables

The aorta was catheterized via the left carotid artery for continuous measurement of mean arterial pressure (MAP, Gould-Statham pressure transducers P23ID, Elk Grove, IL) and intermittent arterial blood gas analysis (Rapidlab 860, Bayer AG, Germany). Cardiac output (CO) and systemic vascular resistance (SVR) were determined via transpulmonary thermodilution (PiCCO 4.2 non US, PULSION Medical Systems, Munich, Germany) at the end of each intervention. Arterial oxygen content and systemic oxygen delivery (DO_2_) were calculated subsequently. Heart rate (HR) was continuously measured by electrocardiography (Powerlab, ADInstruments, Castle Hill, Australia).

#### Gastric and oral mucosal oxygenation and perfusion

Microcirculatory oxygenation (μHbO_2_) and perfusion (μflow) of the gastric and oral mucosa were continuously assessed by tissue reflectance spectrophotometry and laser Doppler flowmetry (O2C, LEA Medizintechnik, Gießen, Germany), as described previously [16]. Briefly, white light (450–1000 nm) and laser light (820 nm, 30 mW) were transmitted to the tissue of interest via a microlight guide and the reflected light was analyzed. The wavelength-dependent absorption and overall absorption of the applied white light can be used to calculate the percentage of oxygenated hemoglobin (μHbO_2_). Due to the Doppler effect, magnitude and frequency distribution of changes in wavelength are proportional to the number of blood cells multiplied by the measured mean velocity (μvelo) of these cells. This product is proportional to flow (μflow) and expressed in arbitrary perfusion units (aU). Hence, this method allows assessment and comparison of oxygenation and perfusion of the examined region at the same time. Since light is totally absorbed in vessels with a diameter > 100 μm, only microvascular oxygenation of nutritive vessels of the mucosa is measured. The biggest fraction of the blood volume is stored in venous vessels; therefore, mainly postcapillary oxygenation is measured which represents the critical partial pressure of oxygen (PO_2_) for ischemia. One flexible light guide probe was placed in the mouth facing the buccal side of the oral mucosa, and a second probe was introduced into the stomach via an orogastric tube. Online evaluation of the signal quality throughout the experiments allowed verification of the correct position of the probe tip. The μHbO_2_ and μflow values reported are the means of the last 5 min (150 spectra, 2 s each) of the respective intervention under steady-state conditions. The non-traumatic access to the gastric mucosa allowed the determination of mucosal microcirculation in the absence of surgical stress. This is particularly desirable with respect to the marked alterations that surgical stress exerts on splanchnic circulation. In this situation, reflectance spectrophotometry reliably detects even clinically asymptomatic reductions in μHbO_2_ [17] and highly correlates with the morphologic severity and extent of gastric mucosal tissue injury [18].

#### Oral mucosal microcirculation—videomicroscopy

Microcirculatory perfusion of the oral, buccal mucosa was consecutively measured by incident dark field (IDF) imaging (CytoCam, Braedius Medical, Huizen, Netherlands) as described elsewhere [19]. Briefly, illumination is provided by light-emitting diodes at a wavelength of 530 nm, the isobestic point for deoxy- and oxyhemoglobin, and directed towards the oral mucosa. The reflected and scattered light is filtered and pictures red blood cells in capillaries. All videos were obtained by the same operator and stored anonymized for blinded analysis. The microcirculation was measured according to the second consensus on the assessment of sublingual microcirculation [20]. To assess perfusion, a semiquantitative scoring method, the microcirculatory flow index (MFI), was used to characterize microcirculatory flow as “no flow,” “intermittent flow,” “sluggish flow,” and “continuous flow” [21]. The total vessel density (TVD), including perfused and non-perfused microvessels, and perfused vessel density (PVD), including perfused microvessels only, were analyzed using dedicated software (MicroCirculation Analysis software, Braedius Medical, Huizen, Netherlands) [22]. The ratio PVD/TVD was used to express the proportion of perfused vessels (PPV). Only vessels with a diameter smaller than 20 μm were included in the analysis, so the PVD represents the functional capillary density, considered to be the main determinant of microcirculatory blood supply [23].

##### Intestinal barrier function

Sucrose and xylose plasma levels were used to assess gastric and intestinal barrier function, respectively. Sucrose (D-Sucrose, Carl Roth, Karlsruhe, Germany) was infused into the stomach (1.66 g·kg^−1^) via an orogastric tube. Under physiological conditions, sucrose does not pass intact gastric mucosa and does not undergo any enzymatic reduction in the stomach [24]. Right after leaving the gastric region into the small intestine, ingested sucrose is rapidly degraded by sucrose-isomaltase into monosaccharides. When gastric mucosal barrier function is disturbed, sucrose can pass over the mucosa into the blood. Once inside plasma, it does not undergo any enzymatic reduction. Sucrose plasma levels can therefore be used to assess gastric mucosal barrier function [24]. Elevated plasma concentrations of xylose, a poorly metabolizable pentose sugar, are indicative of damage to the barrier of the small intestine [25].

Blood samples were collected under baseline conditions and at the end of the experiment. The collected samples were prepared as previously described [26]. Briefly, blood samples were stored in small tubes (Vacutainer K2E EDTA 18.0 mg, Plymouth, UK) and plasma was separated via centrifugation at 0 °C and 3.000 rcf for 15 min (Rotina 420R, Hettich Zentrifugen, Mülheim a.d. R., Germany). Cold extraction solvent mixture containing 10 μM ribitol (Adonitol, Carl Roth, Karlsruhe, Germany) as internal standard, acetone (Aceton, Carl Roth, Karlsruhe, Germany), and isopropanol (2-Propanol, Carl Roth, Karlsruhe, Germany) at a ratio of 2:1 was freshly prepared before use, and 0.4 ml was added to each sample containing 30 μl plasma. Samples were shaken for 5 min at 4 °C prior to centrifugation at 20.800 rcf for 2 min. The liquid supernatant was collected, degassed with a gentle stream of nitrogen (Stickstoff verdichtet, Linde AG, Pullach, Germany), and stored at − 80 °C for later analysis. After resuspension, aliquots of the extracts were dried using a speed vacuum concentrator, measured in two technical replicates each by gas chromatography-mass spectrometry (GC-MS), and analyzed using an appropriate software as described elsewhere [26]. Results are presented as relative amount per microliter plasma.

### Vasopressin receptor blockade

Vasopressin receptor blockade was performed as published previously [11] via bolus application of the selective V1A receptor antagonist [Pmp^1^, Tyr (Me)^2^]-Arg^8^-Vasopressin (Peptanova, Sandhausen, Germany, 35 μg/kg in 20 ml, i.v.), which is known to effectively inhibit V1A receptor response to AVP for > 3 h [27]. Complete receptor blockade was confirmed by administration of 250 mU vasopressin ([Arg^8^]-Vasopressin, Peptanova, Sandhausen, Germany, AVP) at the end of the experiment (groups C and VB). This dose increased MAP by about 20 mmHg without prior V1A receptor blockade but had no effect on MAP after preceding vasopressin receptor blockade.

### Vasopressin plasma levels

In the control group and 30 min after each dose escalation of AVP, blood samples were collected (Vacutainer K2E EDTA 18.0 mg, Plymouth, UK) and plasma was separated via centrifugation and stored at − 20 °C for later analysis by an external laboratory (Labor Limbach, Heidelberg, Germany). As AVP plasma levels during vasopressin blockade are not of informative value, they were not measured.

### Experimental protocol

**Fig. 1:**
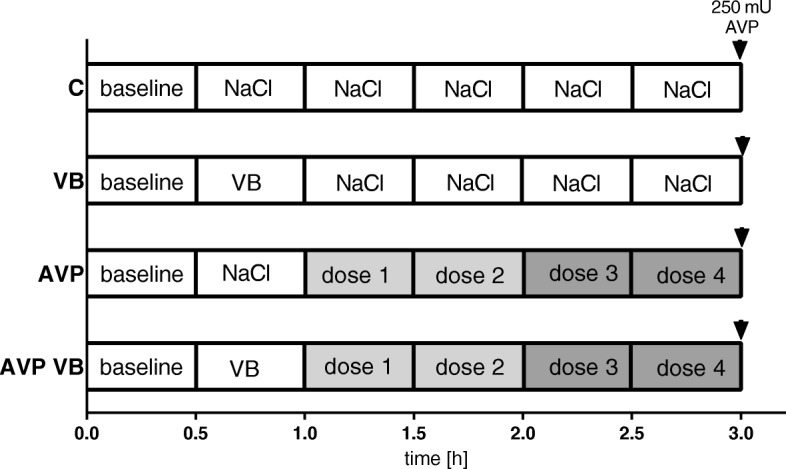
Experimental protocol. Time control treatment with application of normal saline (C) or sole V1A receptor blockade (VB). Application of vasopressin with dose escalation every 30 min in V1A receptor patent dogs (AVP) or with prior application of a V1A receptor inhibitor (AVP VB). Dose escalation of vasopressin: dose 1, 0.001 ng/kg/min; dose 2, 0.01 ng/kg/min; dose 3, 0.1 ng/kg/min; dose 4, 1 ng/kg/min. Doses 1 and 2 represent a subclinical, ultra-low dosage; doses 3 + 4 are comparable to common, clinical low dose AVP application. VB = [Pmp^1^, Tyr (Me)^2^]-Arg^8^-Vasopressin, 35 μg/kg in 20 ml, i.v.; ▼ = 250 mU AVP

After instrumentation, 30 min was allowed to establish steady-state conditions and baseline values were recorded before the animals were randomized to the respective protocol (Fig. 1). Steady-state conditions were defined as stability of hemodynamic variables as well as ventilation parameters. Subsequently, saccharides were administered as described above.

The investigators were blinded concerning the applied drug during the experiment and later analysis. All syringes were specifically prepared for each dog and experiment by a third person and had a similar appearance.

#### Control experiment (C)

As time control experiment, only the vehicle (20 ml NaCl 0.9%) was applied, 30 min later followed by an i.v. infusion of NaCl 0.9% (1 ml/kg/h) for 2 h. To mimic the AVP dose escalation scheme, every 30 min, the syringes were changed.

#### Vasopressin (AVP)

To study the effects of AVP without prior V1A blockade, an i.v. bolus of 20 ml NaCl 0.9% was administered. Afterwards, vasopressin was infused with 0.001 ng/kg/min. Every 30 min, the dose was increased to 0.01 ng/kg/min, 0.1 ng/kg/min, and 1 ng/kg/min. 0.001 ng/kg/min and 0.01 ng/kg/min are referred to as ultra-low dose. For every dose escalation step, a new prefilled syringe was used with constant perfusion rate of 1 ml/kg/h and correspondingly higher AVP concentration.

#### V1A blockade (VB)

To study the effect of sole V1A receptor blockade, after baseline conditions, V1A receptor blockade was initiated as described above. Thirty minutes after application of the receptor blocker, NaCl 0.9% was infused i.v. for 2 h with intermittent syringe exchange as described above.

#### Vasopressin with prior V1A receptor blockade (AVP VB)

To analyze the effects of the V1A receptor in the context of increasing doses of AVP, V1A receptor blockade was initiated as described above. Thirty minutes thereafter, AVP was infused i.v. starting with 0.001 ng/kg/min. Every 30 min, the concentration was increased to 0.01 ng/kg/min, 0.1 ng/kg/min, and 1 ng/kg/min as described above.

In all four types of treatment, blood samples were taken every 30 min for blood gas analysis.

### Statistical analysis

Data for analysis were obtained during the last 5 min of baseline and intervention periods under steady-state conditions. All data are presented as absolute values of mean ± standard deviation (mean ± SD) for six dogs. Normal data distribution was assessed in Q-Q plots (IBM SPSS Statistics, International Business Machine Corp., USA). Differences within and between the treatments were tested using a two-way analysis of variance for repeated measurements (ANOVA) and the Dunnett test as a post hoc test. Differences between treatments AVP and AVP VB were tested using a two-way analysis of variance for repeated measurements (ANOVA) followed by the Bonferroni post hoc test (GraphPad Prism version 6.05 for Windows, GraphPad Software, La Jolla, CA, USA). Differences were considered statistically significant for *p* < 0.05. An a priori power analysis (G*Power Version 3.1.9.2) revealed a power of > 0.8 for detection of differences between the different treatments with *n* = 6 in 4 groups, repeated measurements, α < 0.05 and η^2^ of 0.5 (calculated from previous experiments).

## Results

**Fig. 2:**
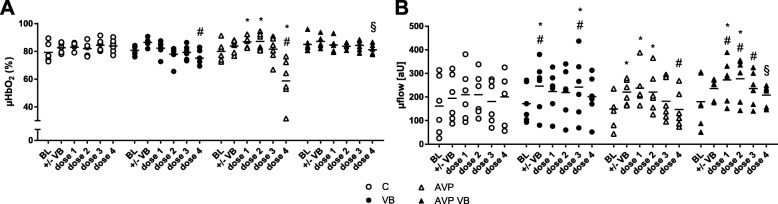
Gastric microcirculation. Gastric microcirculatory oxygenation (μHbO_2_, **a**) and gastric microcirculatory flow (μflow, **b**) in anesthetized dogs in time control experiment (C), with sole V1A receptor blockade (VB) and with AVP dose escalation (dose 1, 0.001 ng/kg/min; dose 2, 0.01 ng/kg/min; dose 3, 0.1 ng/kg/min; dose 4, 1 ng/kg/min) with (AVP VB) or without (AVP) V1A receptor blockade. Data are presented as individual values + mean for *n* = 6 dogs; *p < 0.05 vs. baseline; ^#^*p* < 0.05 vs. control treatment (C), 2-way ANOVA for repeated measurements followed by Dunnett’s post hoc test; ^§^*p* < 0.05 for AVP VB vs. AVP, 2-way ANOVA for repeated measurements followed by Bonferroni’s post hoc test

During control conditions (treatment C) gastric μHbO_2_ remained stable throughout the experiment. Application of 0.001 ng/kg/min and 0.01 ng/kg/min AVP (treatment AVP) led to an increase in gastric mucosal oxygenation from 80 ± 7% to 87 ± 4% and 87 ± 6%, respectively. Further dose escalation to 0.1 ng/kg/min did not change μHbO_2_ compared to baseline values (81 ± 9%), whereas 1 ng/kg/min AVP strongly reduced gastric μHbO_2_ to 59 ± 16%. Prior V1A receptor blockade (treatment AVP VB) abolished these differential effects on gastric μHbO_2_ during dose escalation of AVP (Fig. 2). In parallel with gastric μHbO_2_, AVP enhanced gastric μflow from 149 ± 79 aU (baseline) to 237 ± 78 aU (0.001 ng/kg/min AVP) and 221 ± 87 aU (0.01 ng/kg/min AVP); 1 ng/kg/min decreased gastric microcirculatory perfusion compared to the control treatment. Even after V1A receptor blockade, a similar pattern in gastric μflow was observed with increased gastric perfusion during subclinical AVP dose compared to the control treatment. However, V1A receptor blockade abolished the decrease in μflow during 1 ng/kg/min AVP (Fig. 2). Sole V1A receptor blockade led to a prompt, brief increase in gastric μflow from 172 ± 79 to 246 ± 108 aU (treatment VB). AVP did not significantly modulate gastric μvelo (Table 1).

**Table 1:** Micro- and macrocirculatory variables

Parameter	Treatment	Baseline, 0.5 h	± VB, 1.0 h	Dose 1, 1.5 h	Dose 2, 2.0 h	Dose 3, 2.5 h	Dose 4, 3.0 h
Gastric μvelo (aU)	C	20 ± 8	20 ± 6	20 ± 5	20 ± 5	18 ± 5	20 ± 6
	AVP	19 ± 5	22 ± 3	23 ± 4	22 ± 5	20 ± 5	17 ± 4
	VB	20 ± 5	23 ± 6	22 ± 5	22 ± 6	23 ± 8^#^	22 ± 7
	AVP VB	20 ± 6	20 ± 2	23 ± 4	23 ± 5	21 ± 4	21 ± 3^§^
Gastric rHb (aU)	C	60 ± 9	58 ± 11	57 ± 10	57 ± 12	57 ± 10	56 ± 14
	AVP	54 ± 15	57 ± 12	57 ± 12	59 ± 13	56 ± 11	47 ± 14^*#^
	VB	58 ± 6	59 ± 10	55 ± 10	55 ± 10	54 ± 11	51 ± 10^*^
	AVP VB	60 ± 13	60 ± 8	57 ± 8	59 ± 10	59 ± 13	57 ± 11^§^
Oral μvelo (aU)	C	25 ± 10	24 ± 8	21 ± 7	22 ± 4	24 ± 10	21 ± 4
	AVP	22 ± 6	19 ± 3	23 ± 8	21 ± 5	19 ± 3	16 ± 2
	VB	26 ± 3	29 ± 9	25 ± 5	24 ± 5	24 ± 4	24 ± 3
	AVP VB	25 ± 7	25 ± 6	23 ± 5	25 ± 7	23 ± 3	26 ± 8
Oral rHb (aU)	C	94 ± 10	90 ± 8	87 ± 10^*^	89 ± 6	88 ± 6	89 ± 6
	AVP	91 ± 3	88 ± 5	89 ± 6	89 ± 5	83 ± 5^*^	72 ± 9^*#^
	VB	94 ± 6	95 ± 9	91 ± 6	92 ± 7	91 ± 6	88 ± 8
	AVP VB	95 ± 7	93 ± 6	91 ± 7	92 ± 7	91 ± 8^§^	94 ± 8^§^
TVD (mm/mm^2^)	C	19 ± 1.6	19 ± 2.1	20 ± 1.5	20 ± 1.0	20 ± 1.5	20 ± 2.1
	AVP	21 ± 1.3	20 ± 1.3	20 ± 2.1	20 ± 1.2	20 ± 2.2	18 ± 2.7^*^
	VB	20 ± 0.9	20 ± 1.9	19 ± 1.3^*^	18 ± 1.3^*#^	19 ± 1.6	20 ± 1.3
	AVP VB	19 ± 1.1	19 ± 1.2	19 ± 1.8	20 ± 2.3	19 ± 1.6	20 ± 1.2
PPV (%)	C	56 ± 8.9	49 ± 16.7	49 ± 19.2	53 ± 9.6	57 ± 8.8	56 ± 8.2
	AVP	61 ± 11.0	62 ± 8.0^#^	60 ± 12.9	51 ± 8.1	43 ± 20.8	32 ± 14.6^*#^
	VB	63 ± 5.9	52 ± 13.9	49 ± 17.8	52 ± 12.2	53 ± 12.6	54 ± 16.5
	AVP VB	59 ± 7.4	62 ± 9.5	49 ± 8.7	56 ± 8.5	53 ± 12.6	61 ± 14.7^§^
SV (ml)	C	23 ± 3	23 ± 3	24 ± 3	23 ± 2	23 ± 3	23 ± 3
	AVP	23 ± 2	23 ± 2	23 ± 2	23 ± 2	22 ± 2	22 ± 2
	VB	24 ± 3	25 ± 4^#^	24 ± 3	25 ± 4^#^	26 ± 4^#^	27 ± 4^*#^
	AVP VB	24 ± 4	24 ± 4	26 ± 4^#§^	26 ± 4^*#§^	27 ± 4^*#§^	29 ± 4^*#§^
HR (1/min)	C	121 ± 8	121 ± 6	118 ± 8	119 ± 7	119 ± 6	118 ± 6^*^
	AVP	120 ± 6	120 ± 7	120 ± 7	118 ± 6	116 ± 6^*^	104 ± 10^*#^
	VB	119 ± 4	120 ± 4	119 ± 4	117 ± 4	116 ± 5^*#^	114 ± 6^*#^
	AVP VB	122 ± 9	126 ± 6^*#§^	120 ± 6	119 ± 7	118 ± 8^*^	117 ± 9^*§^
dPmax (mmHg/s)	C	437 ± 21	407 ± 30^*^	403 ± 40^*^	398 ± 37^*^	390 ± 35^*^	395 ± 52^*^
	AVP	447 ± 27	422 ± 32	420 ± 51	415 ± 52^*^	393 ± 59^*^	353 ± 43^*#^
	VB	425 ± 29	425 ± 43	425 ± 45	422 ± 52	428 ± 53^#^	422 ± 53
	AVP VB	450 ± 51	450 ± 72^#^	458 ± 56^#§^	468 ± 56^#§^	477 ± 74^#§^	503 ± 96^*#§^

Micro- and macrocirculatory variables of the different types of treatment—gastric and oral microcirculatory velocity (μvelo), relative hemoglobin amount (rHb), total vessel density (TVD), proportion of perfused vessels (PPV), stroke volume (SV), heart rate (HR), and maximum left ventricular contractility (dPmax). Data are presented as mean ± SD for *n* = 6 dogs

*p < 0.05 vs. baseline

^#^*p* < 0.05 vs. control (C), 2-way ANOVA for repeated measurements followed by Dunnett’s post hoc test

^§^*p* < 0.05 for AVP VB vs. AVP, 2-way ANOVA for repeated measurements followed by Bonferroni’s post hoc test

**Fig. 3:**
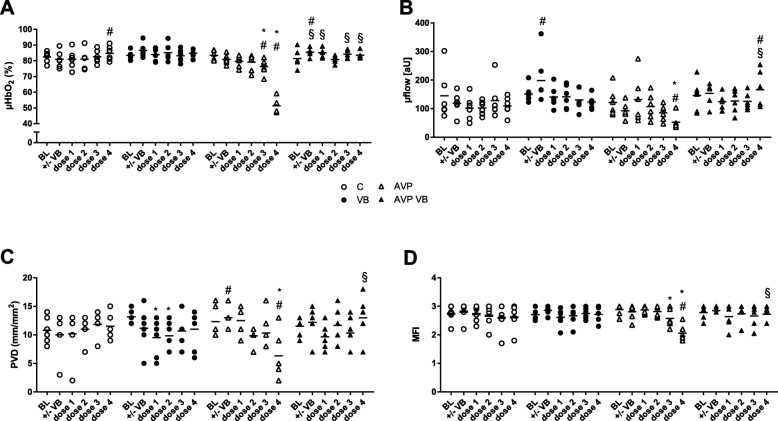
Oral microcirculation. Oral microcirculatory oxygenation (μHbO_2_, **a**), flow (μflow, **b**), perfused vessel density (PVD, **c**), and microvascular flow index (MFI, **d**) in anesthetized dogs in time control experiment (C), with sole V1A receptor blockade (VB) and with AVP dose escalation (dose 1, 0.001 ng/kg/min; dose 2, 0.01 ng/kg/min; dose 3, 0.1 ng/kg/min; dose 4, 1 ng/kg/min) with (AVP VB) or without (AVP) V1A receptor blockade. Data are presented as individual values + mean for *n* = 6 dogs; *p < 0.05 vs. baseline; ^#^*p* < 0.05 vs. C, 2-way ANOVA for repeated measurements followed by Dunnett’s post hoc test; ^§^*p* < 0.05 for AVP VB vs. AVP, 2-way ANOVA for repeated measurements followed by Bonferroni’s post hoc test

In contrast to gastric microcirculatory oxygenation, ultra-low doses of AVP (0.001 ng/kg/min and 0.01 ng/kg/min) did not increase oral μHbO_2_. A reduction in oral μHbO_2_ to 76 ± 5% (0.1 ng/kg/min AVP) and 51 ± 5% (1 ng/kg/min AVP) was observed compared to baseline value (83 ± 3%) in the control treatment (treatment C). V1A receptor blockade abolished this decrease in oral μHbO_2_ (baseline 81 ± 6%; 0.1 ng/kg/min AVP 84 ± 2%; 1 ng/kg/min AVP 84 ± 2%; Fig. 3). AVP dose-dependently modulated oral μflow with a significant decline with 1 ng/kg/min AVP to 53 ± 25 aU compared to its baseline (122 ± 49 aU) and the control treatment (110 ± 32 aU, treatment C), which was undetectable with prior V1A receptor blockade (Fig. 3). A transient increase in oral μflow and μHbO_2_ was observed directly after vasopressin receptor inhibition (treatment VB). The dose-dependent reduction in overall oral microcirculatory perfusion was accompanied by a similar decline in oral capillary density (TVD), overall capillary perfusion (PVD, PPV), and flow quality (MFI) (Fig. 3, Table 1). V1A receptor blockade abolished the vasopressin-induced depression of capillary perfusion.

**Fig. 4:**
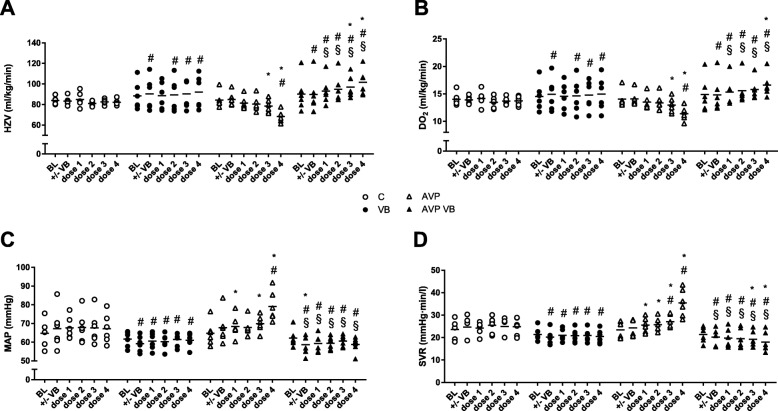
Hemodynamic variables. Cardiac output (CO, **a**), systemic oxygen delivery (DO_2_, **b**), mean arterial pressure (MAP, **c**), and systemic vascular resistance (SVR, **d**) in anesthetized dogs in time control experiment (C), with sole V1A receptor blockade (VB) and with AVP dose escalation (dose 1, 0.001 ng/kg/min; dose 2, 0.01 ng/kg/min; dose 3, 0.1 ng/kg/min; dose 4, 1 ng/kg/min) with (AVP VB) or without (AVP) V1A receptor blockade. Data are presented as individual values + mean for *n* = 6 dogs; *p < 0.05 vs. baseline; ^#^*p* < 0.05 vs. C, 2-way ANOVA for repeated measurements followed by Dunnett’s post hoc test; ^§^*p* < 0.05 for AVP VB vs. AVP, 2-way ANOVA for repeated measurements followed by Bonferroni’s post hoc test

Dose escalation of AVP led to an increase in afterload with a dose-dependent rise in SVR and MAP (Fig. 4) and a reduced HR at constant SV (stroke volume) (Table 1). This was associated with a decrease in CO from 84 ± 8 ml/kg/min to 78 ± 6 ml/kg/min (0.1 ng/kg/min AVP) and 68 ± 6 ml/kg/min (1 ng/kg/min AVP), respectively (Fig. 4). By contrast, during V1A receptor blockade, AVP dose-dependently increased CO and SV in parallel compared to its baseline, sham treatment (treatment C) and AVP treatment without prior receptor blockade (treatment AVP), while SVR significantly declined (Fig. 4). V1A receptor blockade led to an increase in plasma lactate levels during the course of the experiment compared to baseline values. However, these values at no time exceed values during control treatment (Table 2).

**Table 2:** Metabolic variables

Parameter	Treatment	Baseline, 0.5 h	± VB, 1.0 h	Dose 1, 1.5 h	Dose 2, 2.0 h	Dose 3, 2.5 h	Dose 4, 3.0 h
P_a_O_2_ (mmHg)	C	149 ± 6	152 ± 10	155 ± 6	153 ± 9	158 ± 7	159 ± 6^*^
	AVP	152 ± 7	156 ± 11	149 ± 4	154 ± 7	150 ± 5	153 ± 4
	VB	152 ± 10	157 ± 13	155 ± 14	157 ± 10	159 ± 7	158 ± 6
	AVP VB	149 ± 9	150 ± 7	162 ± 7^*§^	163 ± 14^*#^	158 ± 7	161 ± 9^*^
P_a_CO_2_ (mmHg)	C	36 ± 2	36 ± 2	36 ± 2	37 ± 2	36 ± 2	38 ± 2^*^
	AVP	35 ± 1	36 ± 1	36 ± 1	37 ± 1^*^	37 ± 1^*^	37 ± 1
	VB	36 ± 2	36 ± 1	36 ± 1	37 ± 2	37 ± 1	38 ± 2^*^
	AVP VB	36 ± 1	37 ± 2	37 ± 2	36 ± 1	37 ± 1	37 ± 1
S_a_O_2_ (%)	C	98 ± 0.2	98 ± 0.3	99 ± 0.2	98 ± 0.3	99 ± 0.2	99 ± 0.2
	AVP	99 ± 0.2	99 ± 0.3	98 ± 0.1	99 ± 0.2	98 ± 0.2	99 ± 0.1
	VB	99 ± 0.3	99 ± 0.4	99 ± 0.4	99 ± 0.3	99 ± 0.2	99 ± 0.2
	AVP VB	98 ± 0.3	98 ± 0.3	99 ± 0.2	99 ± 0.4	99 ± 0.2	99 ± 0.2
Hct (%)	C	37.9 ± 2.1	37.9 ± 2.0	37.8 ± 2.0	37.7 ± 2.1	37.5 ± 2.1	37.7 ± 2.1
	AVP	37.7 ± 1.2	37.6 ± 1.1	37.6 ± 1.2	37.5 ± 1.2	37.3 ± 1.1	37.6 ± 1.1
	VB	37.2 ± 1.9^#^	37.3 ± 2.0^#^	37.2 ± 2.1^#^	37.0 ± 2.0^#^	36.8 ± 1.9^*#^	36.6 ± 1.9^*#^
	AVP VB	37.4 ± 1.7^#^	37.4 ± 1.7^#^	37.2 ± 1.8^#§^	37.0 ± 1.8^*#§^	36.9 ± 1.8^*#§^	36.9 ± 1.7^*#§^
Hb (g/dl)	C	12.3 ± 0.7	12.3 ± 0.7	12.3 ± 0.6	12.2 ± 0.7	12.2 ± 0.7^*^	12.3 ± 0.7
	AVP	12.3 ± 0.4	12.2 ± 0.4	12.2 ± 0.4	12.2 ± 0.4	12.1 ± 0.4	12.2 ± 0.4
	VB	12.1 ± 0.6^#^	12.1 ± 0.7^#^	12.1 ± 0.7^#^	12.0 ± 0.7^#^	12.0 ± 0.6^#^	11.9 ± 0.6^*#^
	AVP VB	12.1 ± 0.6^#^	12.2 ± 0.5^#^	12.1 ± 0.6^#§^	12.0 ± 0.6^#§^	12.0 ± 0.7^*#§^	12.0 ± 0.6^*#§^
pH	C	7.38 ± 0.02	7.37 ± 0.02	7.36 ± 0.03^*^	7.36 ± 0.03^*^	7.36 ± 0.03^*^	7.35 ± 0.03^*^
	AVP	7.39 ± 0.01^#^	7.39 ± 0.02^#^	7.38 ± 0.01^#^	7.37 ± 0.02^*#^	7.37 ± 0.01^*^	7.37 ± 0.01^*#^
	VB	7.37 ± 0.01	7.37 ± 0.01	7.36 ± 0.01	7.35 ± 0.03^*^	7.36 ± 0.02^*^	7.35 ± 0.02^*^
	AVP VB	7.39 ± 0.02	7.37 ± 0.02	7.37 ± 0.03^*^	7.36 ± 0.03^*^	7.35 ± 0.03^*^	7.36 ± 0.03^*^
HCO_3_^−^ (mmol/l)	C	20.5 ± 0.5	20.2 ± 0.6	20.1 ± 0.9	20.1 ± 0.7	20.0 ± 0.3	20.5 ± 0.3
	AVP	20.7 ± 0.6	21.0 ± 0.6^#^	20.8 ± 0.7^#^	20.8 ± 0.6^#^	20.8 ± 0.8^#^	20.5 ± 0.5
	VB	20.4 ± 0.5	20.3 ± 0.4	20.2 ± 0.6	19.9 ± 1.0	20.0 ± 1.0	20.3 ± 1.1
	AVP VB	21.0 ± 1.1	20.9 ± 1.1^#^	20.5 ± 1.2	20.2 ± 1.4^*^	20.0 ± 1.4^*^	20.2 ± 1.3^*^
Lactate (mmol/l)	C	1.5 ± 0.9	1.9 ± 1.2^*^	2.2 ± 1.4	2.1 ± 1.2	2.0 ± 1.0	1.9 ± 0.8
	AVP	1.0 ± 0.5	1.2 ± 0.6^#^	1.3 ± 0.5^#^	1.3 ± 0.5^#^	1.3 ± 0.5^#^	1.3 ± 0.5
	VB	0.9 ± 0.4	1.0 ± 0.3^#^	1.2 ± 0.5^#^	1.6 ± 1.1^*^	1.7 ± 1.1^*^	1.7 ± 0.9^*^
	AVP VB	0.9 ± 0.4^#^	1.1 ± 0.4^#^	1.6 ± 0.7^*^	2.0 ± 1.2^*^	2.2 ± 1.3^*§^	2.1 ± 1.2^*^

Metabolic variables of the different types of treatment—arterial oxygen partial pressure (P_a_O_2_), carbon dioxide partial pressure (P_a_CO_2_), arterial oxygen saturation (S_a_O_2_), hematocrit (Hct), hemoglobin concentration (Hb), pH, bicarbonate (HCO_3_^−^), and lactate plasma levels. Data are presented as mean ± SD for *n* = 6 dogs

*p < 0.05 vs. baseline

^#^*p* < 0.05 vs. control (C), 2-way ANOVA for repeated measurements followed by Dunnett’s post hoc test

^§^*p* < 0.05 for AVP VB vs. AVP, 2-way ANOVA for repeated measurements followed by Bonferroni’s post hoc test

### Vasopressin plasma concentration

**Fig. 5:**
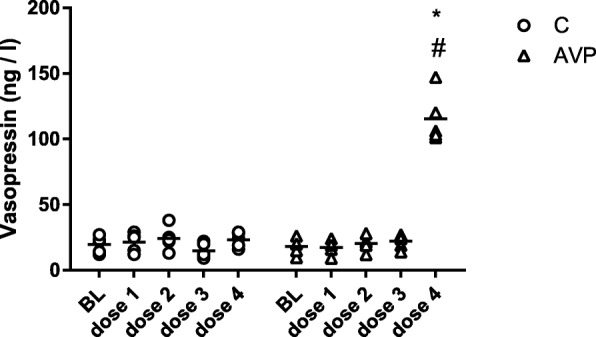
Vasopressin plasma levels. Vasopressin plasma levels during baseline conditions, and 30 min after each dose escalation of vasopressin (treatment AVP) and its respective time control (treatment C). AVP: dose 1, 0.001 ng/kg/min; dose 2, 0.01 ng/kg/min; dose 3, 0.1 ng/kg/min; dose 4, 1 ng/kg/min. Data are presented as individual values + mean for *n* = 5 dogs; *p < 0.05 vs. baseline, ^#^*p* < 0.05 vs. C, 2-way ANOVA for repeated measurements followed by Bonferroni’s post hoc test

During control treatment, AVP plasma concentrations remained constant (14.9 ± 5.7 ng/l–24.2 ± 7.4 ng/l). 0.001 ng/kg/min–0.1 ng/kg/min AVP did not result in increased AVP plasma concentrations compared to its baseline (baseline, 18.1 ± 6.1 ng/l; 0.001 ng/kg/min, 17.5 ± 5.4 ng/ml; 0.01 ng/kg/min, 20.4 ± 5.8 ng/l; 0.1 ng/kg/min, 22.2 ± 5.6 ng/l). Escalation to 1 ng/kg/min AVP led to a considerable rise in AVP plasma concentration (115.4 ± 19.2 ng/l) compared to the baseline and the control treatment (Fig. 5).

### Intestinal barrier function

Baseline sucrose plasma concentrations did not differ and increased during the course of the experiment with a high variability among all four treatment regimes. However, a significant rise was only observed in treatment AVP VB compared to the control treatment (197 ± 130 rel. amount/μl plasma vs. 127 ± 115 rel. amount/μl plasma). Plasma levels of xylose decreased during control treatment, but AVP and V1A receptor blockade had no impact on xylose plasma concentration (Table 3).

**Table 3:** Sucrose and xylose plasma levels

Parameter	Treatment	Baseline, 0.5 h	Dose 4, 3.0 h
Sucrose (rel. amount/μl plasma)	C	11 ± 17	127 ± 115
	AVP	7 ± 3	103 ± 113
	VB	5 ± 7	94 ± 118
	AVP VB	10 ± 9	197 ± 130^*^
Xylose (rel. amount/μl plasma)	C	37 ± 13	27 ± 7^*^
	AVP	32 ± 12	29 ± 8
	VB	30 ± 5	30 ± 4
	AVP VB	35 ± 7	33 ± 5

Sucrose and xylose plasma levels at baseline conditions and at the end of the experiment. Data are presented as mean ± SD for *n* = 6 dogs

*p < 0.05 vs. baseline, 2-way ANOVA for repeated measurements followed by Bonferroni’s post hoc test

A detailed presentation of micro- and macrovascular parameters and further metabolic and respiratory variables is given in Tables 1 and 2.

## Discussion

This study was performed to investigate dose-dependent effects of exogenous AVP on splanchnic microcirculation. Our main results are that exogenous AVP in concentrations as low as 0.001 ng/kg/min and 0.01 ng/kg/min, designated here as ultra-low dose, increases gastric μHbO_2_ and μflow. Prior V1A receptor blockade abolishes the increase in μHbO_2_ but further improves gastric μflow. Thus, improvement of gastric microcirculatory oxygenation is presumably mediated via V1A receptor, while improvement of regional flow is independent of V1A receptor. Effects of ultra-low dose AVP seem to be region specific, as no effects were observed on oral microcirculation; 1 ng/kg/min AVP strongly reduces V1A receptor-dependent gastric and oral μHbO_2_ and μflow, probably via vasoconstriction and decrease of cardiac output due to increased afterload.

In accordance with the guidelines of the Surviving Sepsis Campaign, AVP is clinically administered with 0.03 U/min [2]. This dosage is considered to be low dose. In a patient weighing 70 kg, this corresponds to 0.7 ng/kg/min. Therefore, the AVP dosages 0.001 ng/kg/min and 0.01 ng/kg/min are assumed to be ultra-low dose, with subclinical concentrations, whereas the dosages 0.1 ng/kg/min and 1 ng/kg/min are within the range of daily clinical routine.

The effects of low-dose AVP (0.1–1 ng/kg/min) on macrohemodynamic variables in our model reflect the known V1A-mediated systemic vasoconstriction [4]. A rapid increase in afterload and slight reduction in heart rate were observed together with a substantial decrease in CO and systemic arterial DO_2_. These macrohemodynamic alterations evoke profound changes in the splanchnic and oral microcirculation. A reduced systemic DO_2_ and a pronounced microcirculatory vasoconstriction, indicated by a reduction in variables of overall local mucosal perfusion (μflow), reduced capillary perfusion (TVD, PVD, PPV), and a worsening of perfusion quality (MFI), led to a pronounced decline in local microcirculatory oxygenation. These results are well in line with observations that AVP reduces splanchnic perfusion during physiological conditions in particular [28]. As the AVP-induced systemic vasoconstriction is mainly mediated via the V1A receptor, we could show that prior receptor blockade abolishes these detrimental effects of low dose AVP on gastric and oral microcirculation. Moreover, inhibition of the V1A receptor increases CO and DO_2_, most likely via reduced SVR leading to a decline in afterload and increased SV. Blockade of the V1A receptor may assign the V2 receptor a more prominent role, especially in the context of exogenous AVP administration. Therefore, the stepwise increase in CO and decrease in SVR with dose escalation of exogenous AVP with prior V1A receptor blockade could be explained by an increased activation of the V2 receptor. Beside its involvement in the regulation of fluid homeostasis, the V2 receptor has been found in extrarenal locations, e.g., endothelial cells [29]. Selective V2 receptor agonists have been shown to increase cardiac output and heart rate and decrease systemic peripheral resistance in dogs [30]. However, the regional-specific expression of the V2 receptor is not fully elucidated. Beside the probable action on the V2 receptor, vasopressin has equal affinity for the oxytocin receptor as oxytocin [31]. Among others, this receptor is located on vascular endothelial cells where it has vasodilatory properties via increasing the constitutive endothelial nitric oxide synthase activity [32]. In addition to the V2- or oxytocin receptor-mediated effects, prior V1A receptor blockade might increase AVP-mediated signaling via the V1B receptor located in the pituitary gland, where it modulates corticotropin secretion [4].

Despite a pronounced alteration of microvascular perfusion and oxygenation, no differences in absorption of sucrose and xylose were observed, indicating no AVP-induced damage to the gastrointestinal mucosal barrier. No increase in xylose plasma level was measured at the end of the protocol after application of vasopressin. As expected, low sucrose plasma concentrations were seen before application of the sugar solution. At the end of the experiment, sucrose plasma levels were much higher. However, this increase failed to reach statistical significance compared to baseline values because of a rather large interindividual variability. The phenomenon that gastrointestinal content with high osmolarity is able to increase mucosal permeability by itself even in the absence of pathologic factors [33] might explain the elevated sucrose plasma levels. Because of these limitations, the results of the absorption test have to be interpreted with care and they do not allow a definitive statement on AVP-mediated modulation of gastric barrier function.

In subclinical, ultra-low dosage, AVP exerts different effects on mucosal oxygenation and perfusion compared to the usual clinical, low-dose application and shows differing impact on oral and gastric microcirculation. AVP led to a slight but significant increase in gastric microvascular oxygenation compared to its baseline; however, no differences were observed when compared to the control group. As the main microcirculatory blood volume is stored postcapillary, μHbO_2_ mainly reflects postcapillary oxygenation [16]. Therefore, an enhanced μHbO_2_ could be caused by an increased oxygen supply or reduced utilization. Systemic oxygen delivery remained unchanged during ultra-low dose AVP, despite a slight increase in systemic vascular resistance. One could assume an increased local microcirculatory oxygen supply, as gastric perfusion increased similar to μHbO_2_. This most likely indicates a redistribution of CO. However, increased local microcirculatory oxygen supply cannot fully explain the enhanced μHbO_2_ as V1A receptor blockade abolished the increase in gastric μHbO_2_ but not in local gastric μflow. This is in accordance with findings that in septic rats, V1A receptor blockade abolished a hypercapnia-induced increase in gastric μHbO_2_ but did not influence local μflow [12]. The gastrointestinal perfusion is subjected to a complex regulation, involving among others vasopressin, nitric oxide, norepinephrine, and the renin-angiotensin axis [34]. Therefore, one can speculate that in the context of V1A receptor blockade, one of these other mediators or the vasodilatory acting V2 receptor might assume a key role in regulation of local perfusion.

Exogenous AVP is capable to reduce tissue oxygen consumption, even in a dose which did not alter microcirculatory blood flow [35], probably via modulation of oxygen extraction or demand. In clinical dosage, AVP strongly reduced tissue PO_2_ and increased total oxygen extraction by the microcirculation [36]. An interaction of AVP with mitochondrial respiration was found, which may account for a reduced cellular oxygen demand. Supplemental AVP and terlipressin during resuscitation preserves renal [37] and cerebral mitochondrial function [38] in a rat model of hemorrhagic shock. Therefore, an AVP-induced, probably V1A receptor-mediated, modulation of cellular oxygen demand may account for the increased μHbO_2_ observed in our study. However, despite being statistically significant, one should not overemphasize these results, as the total increase in gastric microcirculatory oxygenation is rather small.

Our observation that gastric and oral microcirculation show a differential response to exogenous AVP is in accordance to other studies indicating that effects of AVP on local perfusion and vascular tone seem to be differently dependent on the studied region [39–41]. In particular, the sublingual and intestinal mucosa may respond differently, e.g., during septic conditions [42]. Regional-specific effects of AVP and probably a different expression of the AVP receptors may account for the different patterns of μflow in gastric and oral mucosal microcirculation with and without prior V1A blockade. As we measured only gastric and oral and not intestinal microcirculatory perfusion, we cannot exclude that the increased gastric μflow with ultra-low dose AVP happened while gut perfusion was decreased. The stomach receives its blood supply mainly via the coeliac trunk, whereas the gut is supplied via the mesenteric arteries. A pronounced, AVP-mediated mesenteric vasoconstriction with consecutively reduced portal venous flow can lead to a reflective increase in perfusion to the coeliac trunk and its branching vessels [43] to maintain hepatic and concomitant gastric perfusion. Therefore, it is of utmost importance that the effects reported in this study refer to the gastric microcirculation and do not provide any reliable information concerning the effects of ultra-low dose AVP on intestinal microcirculation.

AVP plasma concentrations were measured in the control group and during dose escalation of AVP. No changes in AVP plasma levels were observed in the control group during the experiment. Surprisingly, exogenous AVP in concentrations of 0.001 ng/kg/min to 0.1 ng/kg/min did not significantly alter the plasma levels measured 30 min after dose escalation. This is rather astonishing, as even ultra-low dose AVP significantly modified micro- and microcirculatory variables. The measured plasma levels are comparable to the AVP concentrations measured during mild hypercapnia [11]. Baseline AVP levels measured in this model are rather high, compared to the literature. AVP plasma levels during physiological conditions are in the range of 3.5 ng/l in conscious dogs [44]. The anesthetic regime and hemodynamic alterations during anesthesia might be responsible for the generally increased AVP concentrations. In pentobarbitone anesthetized dogs, baseline AVP levels of 14 ± 2 μU/ml plasma were measured, which equals to 23 ± 3 ng/l [45]. In the present study, the dogs were anesthetized with 1.5 MAC Sevoflurane. Sevoflurane is known to raise AVP plasma levels in a MAC-dependent manner [46]. The increased AVP plasma concentrations may overlay effects of ultra-low dose exogenous vasopressin administration. A major fraction of circulating AVP is bound to thrombocytes. As the measurement of AVP plasma levels in EDTA plasma samples without completely separated platelets did not correct for this, the biological active concentration in vivo might be considerably lower [47]. AVP has a half-life period of 5.2 ± 0.4 min for constant infusion in dogs [45]. Therefore, the measurement of AVP plasma concentrations 30 min after dose escalation most likely reflects steady-state conditions; 1 ng/kg/min AVP led to a substantial increase in AVP plasma levels. Comparable AVP levels were observed in the early phase of a profound hemorrhagic shock in thiopental anesthetized dogs [48]. In septic shock patients, continuous infusion of up to 0.03 U/min increased vasopressin levels from 3.5 ng/l to medians of 79.8 ng/l at 6 h and 106.3 ng/l at 24 h [6].

The present study has several limitations. The sample size of *n* = 6 animals per group seems to be rather small compared to usual sample sizes in non-repetitive experiments, e.g., using rats. However, the crossover design allows sufficient power for small sample sizes, as each animal serves as its own control. Furthermore, the small sample size was sufficient to detect significant differences.

Growing evidence indicates that the overall circulatory status determines the vasopressin effects on micro- and macrocirculation. In a hyperdynamic model of ovine septic shock, vasopressin better preserved splanchnic perfusion and resulted in a lower mucosal-arterial PCO_2_ gap compared to noradrenalin [49]. Incremental doses of continuously infused AVP (0.014–0.229 U/kg/h, which corresponds to 0.4 ng/kg/min–6.4 ng/kg/min) did not further compromise jejunal tissue oxygen tension and oxygen supply in LPS-induced endotoxic pigs [50]. In hypodynamic models of septic shock, where volume status and cardiac output did not meet the enhanced needs, AVP and vasopressin analogs worsened splanchnic hemodynamics [51–53]. In healthy, anesthetized pigs, incremental doses of AVP strongly impaired jejunal microcirculatory oxygen supply and mucosal tissue PO_2_ due to a reduction in microvascular blood flow [28]. Incremental doses of exogenous AVP had only a minor effect on blood pressure in healthy volunteers, whereas it strongly increased blood pressure in patients with severe autonomous failure [54]. In this light, the results of the present study have to be interpreted with care and cannot be transferred to pathologic conditions, as it was conducted in anesthetized but otherwise healthy dogs. Therefore, one may expect different, probably more pronounced effects of ultra-low dose vasopressin during pathologic, e.g., septic conditions. In particular, further research is needed with focus on the effects of ultra-low dose AVP administration on intestinal microcirculation and mitochondrial function during various pathologic conditions, e.g., septic and hemorrhagic shock.

## Conclusion

Exogenous AVP modulates microcirculatory oxygenation and perfusion in a dose-dependent and regional-specific manner. In a clinically used concentration of 0.03 U/min, a substantial and potential detrimental reduction of splanchnic perfusion and oxygenation can be assumed. In contrast, subclinical, ultra-low dose AVP concentrations may have favorable effects on gastric microcirculatory oxygenation and perfusion during physiologic circulatory conditions. During V1A receptor blockade and increasing AVP dosage, the vasodilatory properties of other AVP-activated receptors (V2 receptor, V1B receptor, oxytocin receptor) prevail the AVP-induced vasoconstriction. In the last 15 years, growing attention was given to the microcirculation during the treatment of critically ill patients. In this context, modulation of the AVP metabolism with exceptional ultra-low dose AVP administration may evoke unexpected, but favorable outcomes with regard to gastrointestinal microcirculation.

## Data Availability

All data generated or analyzed during this study are included in this published article.

## Abbreviations

ANOVA: Analysis of variance
aU: Arbitrary unit
AVP: Arginine vasopressin
CO: Cardiac output
DO_2_: Systemic oxygen delivery
etCO_2_: End-expiratory carbon dioxide
F_i_O_2_: Inspiratory fraction of oxygen
GC-MS: Gas chromatography-mass spectrometry
HR: Heart rate
IDF: Incident dark field illumination
MAC: Minimal alveolar concentration
MAP: Mean arterial pressure
MFI: Microcirculatory flow index
PO_2_: Partial pressure of oxygen
PPV: Proportion of perfused vessels
PVD: Perfused vessel density
SV: Stroke volume
SVR: Systemic vascular resistance
TVD: Total vessel density
μflow: Microcirculatory flow
μHbO_2_: Microcirculatory oxygenation
μvelo: Microcirculatory mean velocity
